# 17‐α estradiol ameliorates age‐associated sarcopenia and improves late‐life physical function in male mice but not in females or castrated males

**DOI:** 10.1111/acel.12920

**Published:** 2019-02-10

**Authors:** Michael Garratt, Danielle Leander, Kaitlyn Pifer, Brian Bower, Jonathan J. Herrera, Sharlene M. Day, Oliver Fiehn, Susan V. Brooks, Richard A. Miller

**Affiliations:** ^1^ Department of Pathology University of Michigan Medical School Ann Arbor Michigan; ^2^ Molecular and Integrative Physiology University of Michigan Ann Arbor Michigan; ^3^ Internal Medicine University of Michigan Ann Arbor Michigan; ^4^ Genome Center University of California Davis Davis California; ^5^ Department of Biomedical Engineering University of Michigan Ann Arbor Michigan; ^6^ University of Michigan Geriatrics Center Ann Arbor Michigan

## Abstract

Pharmacological treatments can extend mouse lifespan, but lifespan effects often differ between sexes. 17‐α estradiol (17aE2), a less feminizing structural isomer of 17‐β estradiol, produces lifespan extension only in male mice, suggesting a sexually dimorphic mechanism of lifespan regulation. We tested whether these anti‐aging effects extend to anatomical and functional aging—important in late‐life health—and whether gonadally derived hormones control aging responses to 17aE2 in either sex. While 17aE2 started at 4 months of age diminishes body weight in both sexes during adulthood, in late‐life 17aE2‐treated mice better maintain body weight. In 17aE2‐treated male mice, the higher body weight is associated with heavier skeletal muscles and larger muscle fibers compared with untreated mice during aging, while treated females have heavier subcutaneous fat. Maintenance of skeletal muscle in male mice is associated with improved grip strength and rotarod capacity at 25 months, in addition to higher levels of most amino acids in quadriceps muscle. We further show that sex‐specific responses to 17aE2—metabolomic, structural, and functional—are regulated by gonadal hormones in male mice. Castrated males have heavier quadriceps than intact males at 25 months, but do not respond to 17aE2, suggesting 17aE2 promotes an anti‐aging skeletal muscle phenotype similar to castration. Finally, 17aE2 treatment benefits can be recapitulated in mice when treatment is started at 16 months, suggesting that 17aE2 may be able to improve aspects of late‐life function even when started after middle age.

## 1 INTRODUCTION

With an increased proportion of individuals living to older ages, a greater proportion of the human population suffers from frailty and impaired physical function. Demographic models predict that the number of people living to old ages in high‐income countries will increase (Colby & Ortman, 2017; Robine & Cubaynes, 2017), which presents a potentially substantial burden for healthcare and economic systems. Interventions that can slow age‐related physical decline and improve health later in life would help to ameliorate this burden, while improving the quality of life for elderly adults. Pharmacological treatments are increasingly being recognized as potential methods to slow functional declines during aging in humans (Longo et al., 2015), in addition to reducing the incidence of age‐associated morbidities and neurological decline.

One area of pharmacological research that has already received attention in the context of aging is steroid treatments that seek to redress alterations in circulating sex hormone concentrations that occur during later life. Manipulation of testosterone and estrogens can improve aspects of physical function in the elderly (Horstman, Dillon, Urban, & Sheffield‐Moore, 2012; Stanworth & Jones, 2008), but can also elevate risks of certain diseases, including cancers and cardiovascular disease (Basaria et al., 2010; Chen & Colditz, 2007), potentially because of their strong binding affinity to classical steroid receptors across the body. More recently, other steroids, with lower binding affinities to classical sex hormone receptors, have been suggested as alternative treatments to protect against aging, while lessening side‐effects of diseases linked to classical sex hormone signaling (Gonzalez‐Freire, Diaz‐Ruiz, & Cabo, 2016; Madak‐Erdogan et al., 2016). 17‐α estradiol (17aE2), a less feminizing structural isomer of 17‐β estradiol, has been shown to extend lifespan in male mice (Strong et al., 2016), while also improving glucose tolerance and lowering the abundance of circulating inflammatory cytokines (Garratt, Bower, Garcia, & Miller, 2017; Stout et al., 2016). Effects of 17aE2 on lifespan and metabolism are strongly sex‐specific, with neither lifespan (Strong et al., 2016) nor adult glucose tolerance (Garratt, Bower et al., 2017) detectably affected by 17aE2 in female mice.

While 17aE2 has male‐specific benefits for survival, we have limited understanding of whether these effects extend to functional, pathological, or biochemical age‐associated changes, and whether slowed aging responses outside of survival also differ between males and females. Furthermore, we currently have a poor understanding of what mechanisms underlie sexual dimorphism in response to anti‐aging interventions, observed with 17aE2, but also an increasing number of other pharmacological and genetic interventions (Austad & Fischer, 2016), including reduced IGF1 (Garratt, Nakagawa & Simons, 2017; Holzenberger et al., 2003) and mTORC1 signaling (Garratt, Nakagawa, & Simons, 2016; Lamming et al., 2012; Selman et al., 2009)*.* Our previous research has shown that sex‐specific metabolic responses to 17aE2 in adulthood are linked to the presence of male gonads, such that male‐specific improvements in glucose tolerance are inhibited if males are castrated prior to the onset of treatment (Garratt, Bower et al., 2017; Garratt et al., 2018). However, whether gonadal hormones control the anti‐aging effects of 17aE2, or any other sexually dimorphic anti‐aging manipulation, has not been tested.

In this study, we show that 17aE2 treatment has anti‐aging effects for body weight regulation, muscle weight, and physical function, and that these effects differ strongly between males and females. We used two independent cohorts of mice to probe these effects, while establishing the underlying hormonal causes for the observed sex‐specificity, and to test whether the anti‐aging effects of this treatment can be recapitulated by treatment beginning in middle age. In cohort 1, all mice underwent a brief surgery at 3 months of age, where gonads (testes or ovaries) were either removed (gonadectomy) or exposed but remained in place (sham gonadectomy). These animals then began 17aE2 treatment at 4 months of age, or stayed on a control diet, and were euthanized at 25 months. Animals in cohort 2 did not undergo any surgery and were euthanized at 22 months. The main cohort of animals began 17aE2 treatment at 4 months, while a subset remained on the control diet until 16 months of age, but were then switched to 17aE2 treatment at 16 months of age. This allowed us to test whether a late onset treatment of 17aE2 can also produce functional benefits later in life, and to compare the anti‐aging effects of treatment onset at these two time points.

## 2 RESULTS

### 2.1 17aE2 maintains body weight during aging in both sexes, but has sex‐specific effects on body composition: intact, sham‐operated animals (Cohort 1)

**Figure 1:**
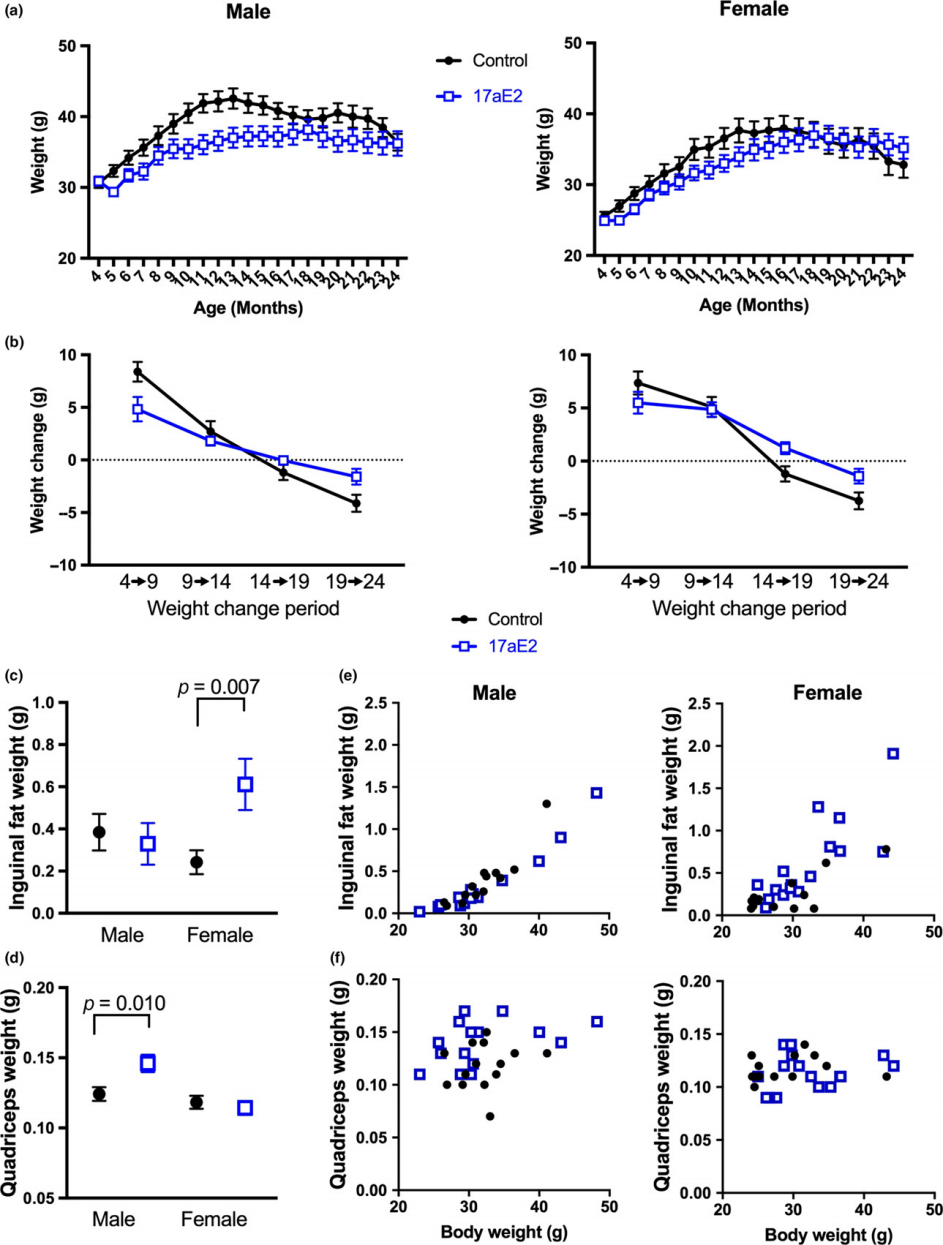
Changes in body weight, skeletal muscle, and subcutaneous fat weight in male and female mice treated with 17aE2. Data presented in (a) show the mean body weights of male and female mice from cohort 1 on control or 17aE2 diets across life, while in (b), the change in weight is shown calculated from two specific time points. Data in (c) and (d) show the weights of inguinal fat and quadriceps at dissection, either plotted against body weight individually for each mouse, or the mean weight of each group. Error bars represent the standard error of the mean (*SEM*). *p* values for quadriceps and inguinal weight were calculated from a Student's *t* test. *N* = 11–20 per group

In the first cohort of mice treated with 17aE2 from 4 months, we recorded body weight monthly across life (Figure 1a). Effects of 17aE2 on body weight differed depending on life‐stage (*p* = 0.001 for the interaction between 17aE2 treatment and time in a repeated measures ANOVA of monthly body weights), but were similar in intact (sham‐operated) animals of both sexes (*p* = 0.32 for the 3‐way interaction between sex, treatment, and time:; *p* = 0.61 for the interaction between sex and treatment; Figure 1b), as previously reported (Strong et al., 2016). 17aE2 reduces weight gain over approximately the first 12 months of life (Figure 1b), as shown by the change in weight between 4 and 9 months in Figure 1b. This presumably reflects the reduction in adiposity that occurs with the onset of 17aE2 treatment (Steyn et al., 2018; Stout et al., 2016). However, we observed that during aging, 17aE2 slows the decline in body weight that occurs over late‐life periods. This is most clearly illustrated by the change in weight between 19 and 24 months of age (Figure 1b). At 25 months, all animals were euthanized, and major organs and fat pads weighed, allowing us to test whether late‐life weight effects were linked to alterations in the weight of specific tissues (see Supporting Information Table S1 for weights of all tissues). In female mice, 17aE2 increased the weight of subcutaneous inguinal fat at 25 months (Figure 1c), apparently contributing to the maintenance of body weight between 19 and 24 months in females. In males, 17aE2 did not significantly alter the weight of inguinal fat (Figure 1c) but led to an increased skeletal muscle weight at 25 months as assessed by quadriceps weight (Figure 1d). This effect of 17aE2 persists whether assessing total muscle weight or muscle weight corrected by body weight (Figure 1d,f) and represents a significant sex‐specific response, in that there was a significant interaction between sex and treatment in an ANCOVA model including body weight as a covariate (Table 1). The effect of 17aE2 on muscle weight was also age‐specific, since quadriceps weight in a subset of animals from cohort 1 euthanized at 12 months of age was not altered by 17aE2 treatment (Supporting Information Figure S1A), and muscle weight of 25‐month‐old control animals was significantly lower than in muscles taken from a set of 6‐month‐old untreated animals of the same strain that were euthanized, dissected, and weighed over the same period (Supporting Information Figure S1B).

**Table 1:** Effect of age, 17aE2 treatment and sex on skeletal muscle and functional traits. *p* values for age effects represent the main effect of age in a 2‐way ANOVA, including both age and sex as variables. *p* values for interaction terms were also calculated from a 2‐way ANOVA, including an effect of treatment (control or treatment) and a second term representing either sex or surgical status. For quadriceps weight, body weight was also included as a continuous covariate in the analysis to account for variation in body weight across mice

	Cohort	Effect of age		Effect of 17aE2	Sex by 17aE2 treatment interaction	Surgery by treatment interaction:Male	Surgery by treatment interaction: Female
		Change	*p* value				
Quadriceps weight	1&2	Decreased	<0.001	Increased in males	0.004	<0.001	0.34
Gastrocnemius fiber size	1	Decreased	0.006	Increased (*p* = 0.010 for both sexes)	0.91		
Angular atrophy of fibers	1	Increased	0.001	Decreased in males	0.030	0.010	0.78
Rotarod Performance	1&2	Decreased	0.006	Increased in males	0.064	0.010	0.80
Centralization of fiber nuclei	1	Increased	0.006	—	—		
Gastrocnemius weight	2	Decreased	0.058	Increased in males	0.041	Not tested	
Grip strength	2	Decreased	<0.001	Increased in males	0.047	Not tested	
Type 2b fiber CSA (gastroc)	2	Decreased	0.037	Increased (*p* = 0.018 for both sexes)	0.24		
Type 2a fiber CSA (gastroc)	2	Unchanged	—	—			
Type 1 fiber CSA (gastroc)	2	Unchanged	—	—			
Type 1 CSA (Soleus)	2	Unchanged	—	—			
Type 2a CSA (Soleus)	2	Unchanged	—	—			

### 2.2 17aE2 maintains skeletal muscle fiber size during aging in male mice: intact animals (Cohorts 1&2)

**Figure 2:**
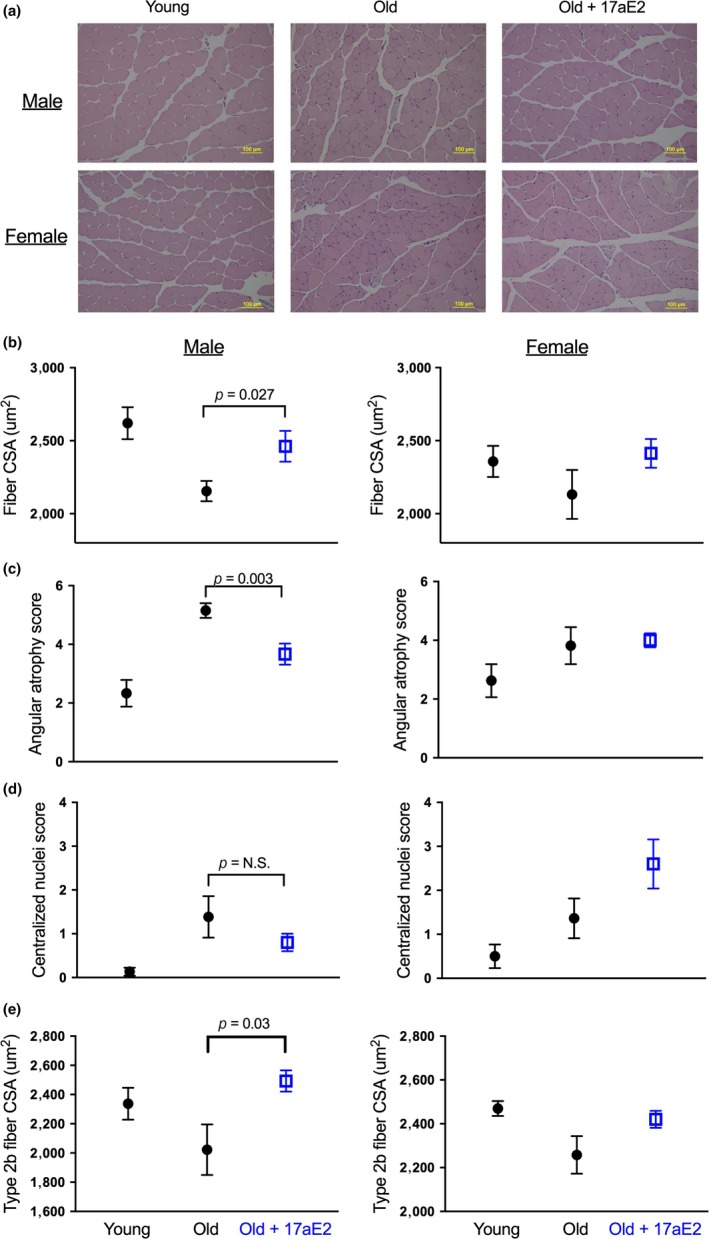
Increased skeletal muscle fiber size and reduced atrophy in 17aE2 treated male mice. (a) Representative images of cross‐sections of gastrocnemius muscles (×20 magnification) from young (6 months), old (25 months), and old mice treated with 17aE2. (b) Average fiber CSA determined from cross‐sections of the gastrocnemius muscle, while (c) and (d) show scores for degree of angular atrophy and centralization of nuclei across different treatment groups in cohort 1 (25 months). (e) Type‐2b fiber CSA from mice in cohort 2 (sampled at 22 months). Error bars represent *SEM*. *p* values are calculated from a Student's *t* test. *N* = 8–15 per group for panels a–d, 5–6 for Figure 2e

To understand whether the delay in sarcopenia represents changes at the level of the individual muscle fibers, we first measured muscle fiber size at 25 months. Fiber cross‐sectional areas (CSA) were measured in gastrocnemius muscles collected from animals in cohort 1 and fixed in 10% buffered formalin immediately at dissection. Compared to samples taken from 6‐month‐old young controls, 25‐month‐old intact control mice showed a reduction in average muscle fiber CSA, an effect that was ameliorated in intact male mice treated with 17aE2 from 4 months (Figure 2a,b; Table 1). Fiber CSA showed a similar response to 17aE2 treatment in intact female mice (Figure 2b; Table 1). We also observed that intact male mice treated with 17aE2 maintained typical muscle fiber morphology during aging and did not present the severe angular deformation of muscle fibers observed in untreated old animals (Hepple, Ross, & Rempfer, 2004; Purves‐Smith, Solbak, Rowan, & Hepple, 2012) (Figure 2c). This represents a sex‐specific response as indicated by the sex by treatment interaction term (Table 1) and the lack of response in this parameter in 17aE2 treated females (Figure 2c). Old animals also showed characteristic accumulation of fibers with central nuclei, a change that was not significantly inhibited by 17aE2 treatment in either sex (Table 1; Figure 2d).

The gastrocnemius muscle is made up of a mix of muscle fiber types, although the large majority are type 2b fast‐twitch muscle fibers. Fast‐twitch muscle fibers typically show the greatest atrophy during aging, with fewer changes observed in slow‐twitch oxidative fibers (Russ, Gregg‐Cornell, Conaway, & Clark, 2012). In the second cohort of mice sampled at 22 months, we assessed whether the effects of 17aE2 on muscle fiber size were fiber type specific, by examining the size of individual muscle fibers of different fiber types within the gastrocnemius muscle. Given the increase in fiber size observed in the gastrocnemius muscles of cohort 1, we also weighed gastrocnemius muscles of animals in cohort 2, which showed that this skeletal muscle was significantly heavier in 17aE2‐treated male mice compared to controls (*p* = 0.006, data are plotted in a subsequent figure (Figure 6)), with no change in females, demonstrating a sex‐specific response (Table 1). 17aE2 treatment increased the CSA of fast‐twitch glycolytic type2b muscle fibers in gastrocnemius muscle (Figure 2e), without affecting the CSA of oxidative type 1 or type 2a fibers, which also did not change significantly with age (Table 1; Supporting Information Figure S2; see Supporting Information Figure S2 for representative images for each muscle fiber type). We also assessed CSA of muscle fibers in the soleus muscle, which is comprised almost entirely of type 1 and type 2a muscle fibers, with type 2b fibers absent (Kammoun, Cassar‐Malek, Meunier, & Picard, 2014). Data from soleus muscles further demonstrated a lack of change in the size of these oxidative skeletal muscle fibers with aging (Supporting Information Figure S3)—consistent with previous reports (Williams, Higgins, & Lewek, 2002)—or 17aE2 treatment (Supporting Information Figure S3), indicating a predominant effect of 17aE2 on fast‐twitch muscle fibers. In cohort 2, we also measured the weight of the quadriceps muscles. Similar to the findings in the 25‐month‐old mice of cohort 1, at 22 months the quadriceps muscle weight was greater in 17aE2 treated male mice than in untreated controls (Supporting Information Figure S1C), although at this age the *p* value did not reach the traditional criterion for statistical significance (*p* = 0.052).

### 2.3 17aE2 treatment improves grip strength and rotarod performance in aging intact male mice (Cohorts 1&2)

**Figure 3:**
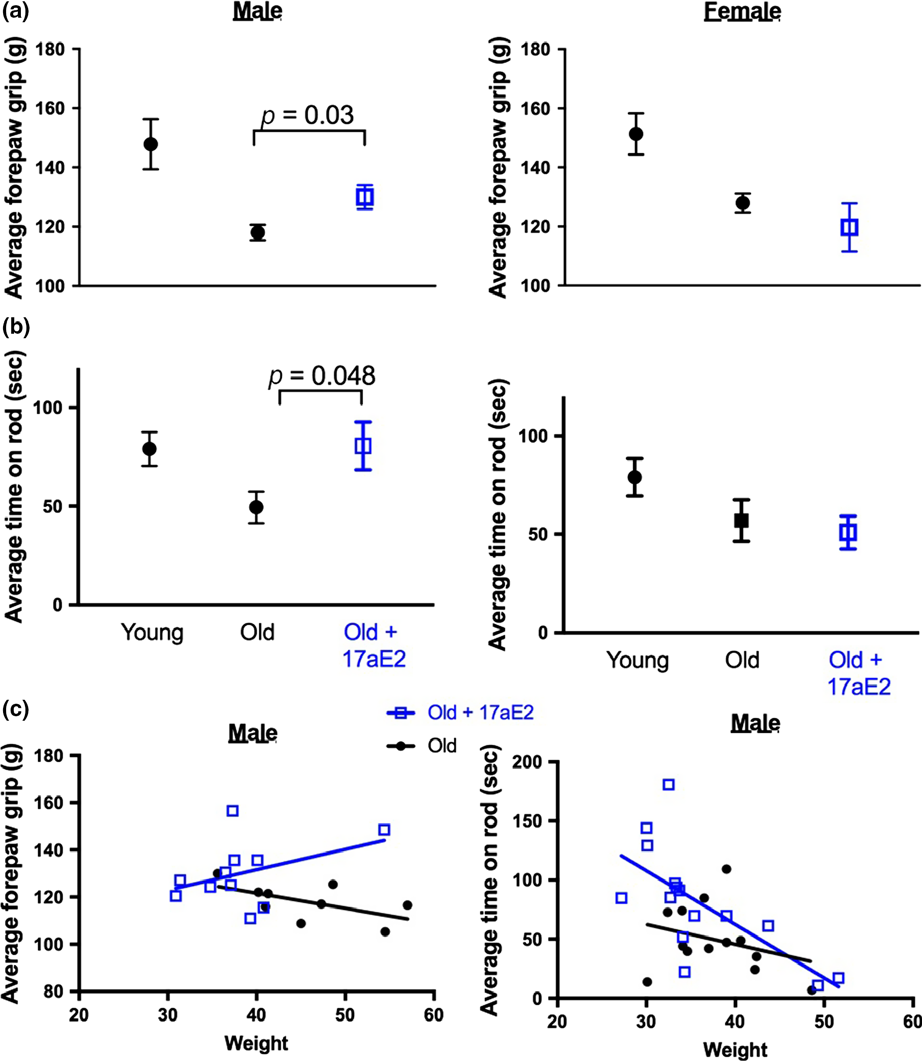
17aE2 increases grip strength and rotarod capacity of aging male mice. (a) Forepaw grip strength in 22‐month‐old male and female mice treated with 17aE2, and young (6 months) and old (22 months) controls (cohort 2, *N* = 8–9 per group for males, 8–29 for females). (b) Rotarod capacity in 24‐month‐old male and female mice treated with 17aE2, young (6 months) and old controls (24 months) (*n* = 15–20 per group). (c) The relationship between grip strength/rotarod capacity and body weight in old male mice, with each dot representing values for an individual mouse. Error bars represent *SEM* and *p* values are calculated from a Student's *t* test

To test whether the effects of 17aE2 treatment on muscle aging are associated with improvements in late‐life physical function, we assessed forepaw grip strength and rotarod performance. Forepaw grip strength was assessed at 22 months in cohort 2 and was lower in these animals than in a comparable set of 6‐month‐old controls (Figure 3a; Table 1). 17aE2 treatment improved male grip strength but had no effect on female grip strength (Figure 3a). We assessed rotarod performance at 24 months of age in cohort 1 using an acceleration protocol where mice were tested for their ability to balance on a progressively accelerating rotarod. The ability of mice to maintain balance on the rod declines with age, while 17aE2 treatment significantly improves balance ability in intact male mice (Figure 3b). Female performance was not affected by 17aE2 treatment, and these sex‐specific effects were also replicated independently in cohort 2 (shown in subsequent Figure 6). We also tested whether differences in performance under these tests could be accounted for by changes in body weight that can occur with 17aE2 treatment. The relationship between grip strength and body weight across male mice was not significant (*p* = 0.79 for effect of body weight as a covariate), suggesting that variation in body weight between groups does not account for the improved grip strength in male mice treated with 17aE2. Rotarod performance was negatively related to body weight (Figure 3c; *p* = 0.002 for effect of body weight as a covariate). When this negative relationship was accounted for by including weight as a covariate, mice treated with 17aE2 still showed improved rotarod performance relative to body weight (Figure 3c), although the *p*‐value for an effect of 17aE2 treatment in cohort 1, when including body weight as a covariate, failed to reach statistical significance (*p* = 0.062). We note that the same relationship between body weight, rotarod performance, and 17aE2 treatment was observed in males in cohort 2 at 22 months. Combining both datasets to increase statistical power revealed a significant effect of 17aE2 treatment in male mice across both cohorts (*p* = 0.015), even when accounting for variation in weight by including body weight as a covariate.

### 2.4 17aE2 generates sexually dimorphic responses in skeletal muscle amino acid abundance (Cohort 1)

**Figure 4:**
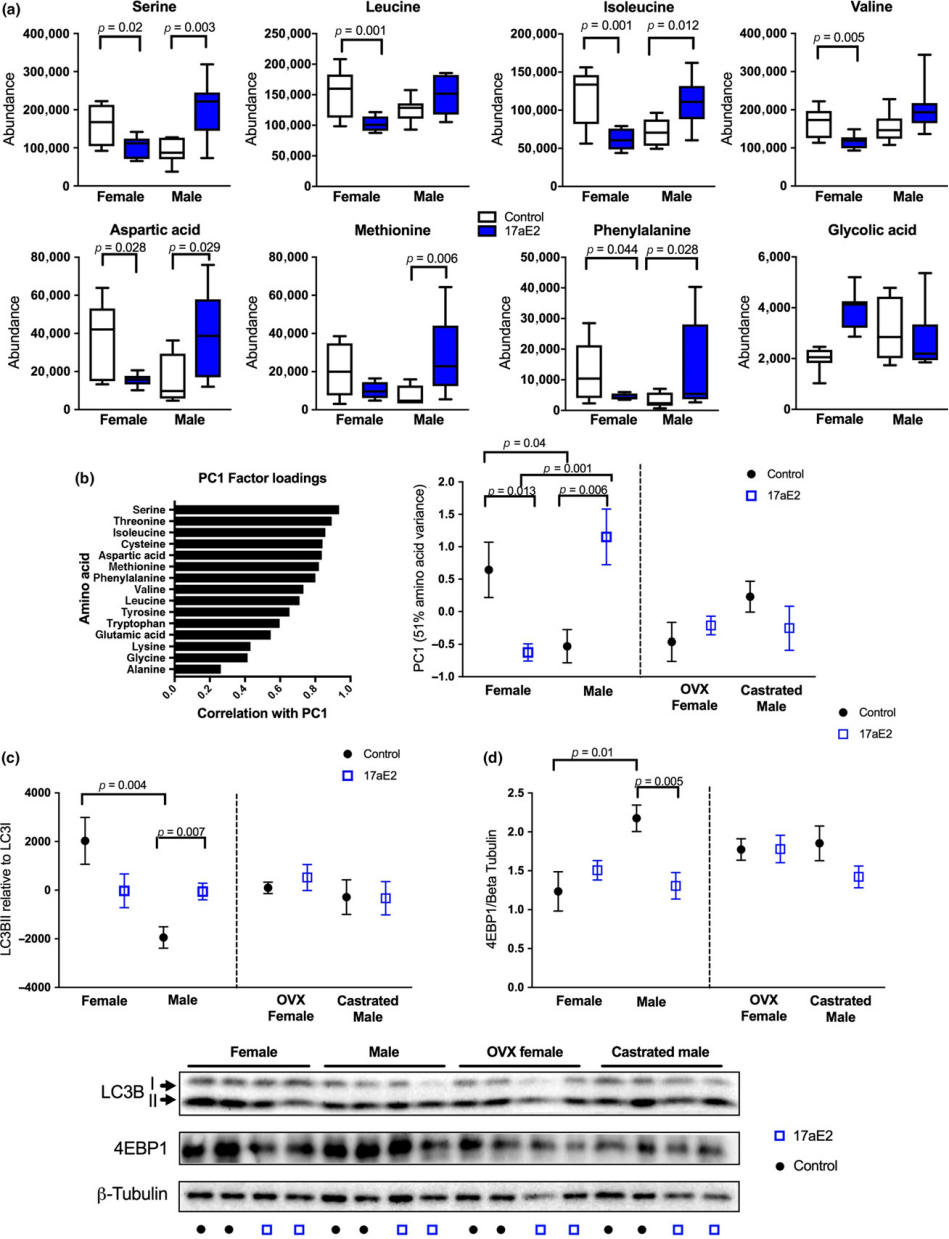
17aE2 causes a sex‐specific amino acid response in quadriceps that is regulated by gonadal hormones. (a) Metabolites showing a sex‐specific treatment response. Box plots tails show min and max values. (b) Principal component analysis showing amino acid factor loadings for PC1 and sex, treatment and surgery scores for PC1. (c) LC3BII and (d) 4EBP1 abundance in mice of different surgical, sex and treatment status, assessed in whole cell muscle homogenate using western blot. Error bars represent *SEM* and *p* values are calculated from a Student's *t* test. *N* = 6–8 per group

To test whether sex‐specific morphological responses to 17aE2 during aging were matched by sex‐specific biochemical changes in muscle, we conducted an untargeted analysis of primary metabolites in quadriceps muscle sampled at 25 months from Cohort 1. Using a 2‐way ANOVA to identify metabolites showing a sex‐specific response to 17aE2 in intact (sham‐operated) animals, we observed 8 metabolites that showed a significantly different response to 17aE2 treatment in each sex after correction for false discovery rate (i.e., a sex by treatment interaction effect: Table 2), seven of which were amino acids, and the other was glycolic acid (Figure 4a; Table 2). Additional analysis of other amino acids detected in this screen showed this was a relatively consistent response in amino acids (Table 2; Supporting Information Figure S4) and reflects an increase in amino acid abundance with 17aE2 treatment in males, but a reduction in females.

**Table 2:** Quadriceps muscle metabolites showing a sex‐specific response to 17aE2 treatment. Sex‐specific metabolites represent those metabolites that show a significant (*p* < 0.05) sex by treatment interaction after correction for FDR. *p* values presented in this table are uncorrected for multiple comparisons

Metabolite	Treatment interaction (*p*‐value 2‐way ANOVA)			Effect of 17aE2 (*p*‐Value Student's *t* test)			
	Sex (intact mice)	Cast (male)	OVX (female)	Male	Female	Cast Male	OVX Female
Sample size control				8	7	8	8
Sample size 17aE2				9	8	8	8
Sex‐specific metabolites							
Isoleucine	0.00003	0.015	0.15	↑0.011	↓0.001		
Serine	0.00011	0.005	0.018	↑0.003	↓0.019		
Aspartic acid	0.0005	0.003	0.002	↑0.019	↓0.009	↓0.087	↑0.066
Leucine	0.00075	0.52	0.029		↓0.001		
Valine	0.001	0.020	0.039	↑0.056	↓0.005		
Glycolic acid	0.00152				↑<0.0001		
Phenylalanine	0.0027	0.010	0.031	↑0.028	↓0.044		
Methionine	0.0027	0.001	0.039	↑0.006			↑0.092
Other amino acids							
Tryptophan	0.030	0.008	0.17	↑0.073			
Threonine	0.006	0.005	0.24	↑0.002			
Lysine	0.021	0.001	0.15	↑0.015		↓0.042	
Glycine	0.018	0.048	0.25	↑0.062			
Glutamic acid	0.12	0.50	0.044		↓0.087		
Cysteine	0.005	0.029	0.054	↑0.072	↓0.032		
Alanine	0.86	0.48	0.76		↑0.093		

Because the abundance of most amino acids is highly correlated, we used principal component analysis to convert the abundance data from all 15 amino acids generated from all samples in cohort 1 into fewer principal components that explained variation across amino acids. This analysis produced one major principal component (PC1) that explained 51% of the variance across the dataset and was significantly correlated with the abundance of all amino acids, although the relationship was strongest with serine and weakest with alanine (Figure 4b), reflecting the strength of treatment responses seen for individual amino acids (Table 2). The second and third principal components extracted in this model only explained 6% and 1% of variation, respectively. There is a strong sex by treatment interaction for PC1 scores in sham‐operated animals (*p* < 0.001). This reflects an elevated abundance of amino acids in intact females on the control diet, but a switch under 17aE2 treatment, with intact males increasing amino acids and females showing a significant reduction (Figure 4b).

### 2.5 Sex‐specific amino acid responses to 17aE2 are dependent on gonadal hormones (Cohort 1)

We used the principal component analysis to test whether the sex‐specific amino acid responses to 17aE2 were dependent on the production of gonadally derived hormones, by comparing metabolite responses to 17aE2 in mice that were gonadectomized at 3 months, prior to 17aE2 treatment, with responses observed in sham‐operated (intact) animals. While intact males show an elevation in amino acids with 17aE2 treatment, this effect is blocked in males that were castrated prior to drug treatment. This is reflected in the lack of response in PC1 to 17aE2 treatment in castrated males (Figure 4b), and the failure of 17aE2 treatment to increase the abundance of any amino acid in castrated males (Table 2). In a 2‐way ANOVA of PC1 scores comparing the effect of surgical status (intact or castrated) and drug treatment (control or 17aE2) in male mice, there is a strong interaction term (*p* = 0.003), further demonstrating that the male response to 17aE2 depends on the presence of male gonads. In females, ovariectomy prior to treatment also blocked the female‐specific declines in amino acids (Figure 4b), and there was an interaction between surgical status and treatment (*p* = 0.011), indicating that the amino acid responses to 17aE2 in intact animals of both sexes were linked to the presence of male and female gonads.

Increased amino acids in muscle may represent a consequence of altered protein synthesis or breakdown, both of which can be regulated by the actions of gonadally derived hormones (Rossetti, Steiner, & Gordon, 2017). To explore whether sexually dimorphic responses to 17aE2 extend to mechanisms regulating protein synthesis and autophagy, we assessed the status of protein substrates involved in autophagy and protein translation in samples taken from a subset of animals in cohort 1 at 12 months of age. Males and females show a strong difference in relative LC3BII to LC3BI levels, a marker of autophagosome formation, with females having greater LC3BII relative to LC3BI, as previously reported (Tao et al., 2018). This could be a consequence of either greater autophagosome formation or slowed autophagic degradation in female mice (Mizushima & Yoshimori, 2007). Importantly, the sex difference is completely lost with 17aE2 treatment, with males and females showing different responses to 17aE2 treatment (sex by treatment interaction term: *p* = 0.002). Specifically, males show an increase in relative LC3BII abundance after 17aE2 treatment (Figure 4c), while LC3BII declines in 17aE2‐treated females. In untreated animals, male castration increases LC3BII, as previously reported (Serra et al., 2013), and female ovariectomy reduces LC3II (Figure 4c). Neither castrated males nor ovariectomized females show a significant change in LC3II levels with 17aE2 treatment. The surgery by treatment interaction test within each sex provides statistical support for a different response to 17aE2 treatment in OVX females compared to intact females (*p* = 0.008), but not in the comparison with castrated to intact males (*p* = 0.18).

We also examined effects of 17aE2 on mTORC1 signaling, a key regulator of protein synthesis that has sexually dimorphic effects on physiology and aging in mice (Lamming et al., 2012). We observed no changes in relative phosphorylation of S6 and 4EBP1, downstream targets of mTORC1 (Supporting Information Figure S5). We also assessed total protein levels of 4EBP1, since genetically engineered over‐expression of 4EBP1 can protect against male‐specific adiposity and dysregulated insulin sensitivity (Tsai et al., 2015). Relative 4EBP1 protein levels are strongly reduced in male mice treated with 17aE2 (Figure 4d), but unaffected by treatment in females, with the sex difference in protein levels seen in animals on the control diet lost with 17aE2 treatment (sex by treatment interaction: *p* = 0.006). The sex difference in 4EBP1 protein levels is also not observed in gonadectomized animals on the control diet (Figure 4d), although these animals show a similar response to intact animals when treated with 17aE2 (surgery by treatment interaction *p* > 0.1 in both sexes).

### 2.6 Functional and structural responses to 17aE2 are blocked by male castration (Cohort 1)

**Figure 5:**
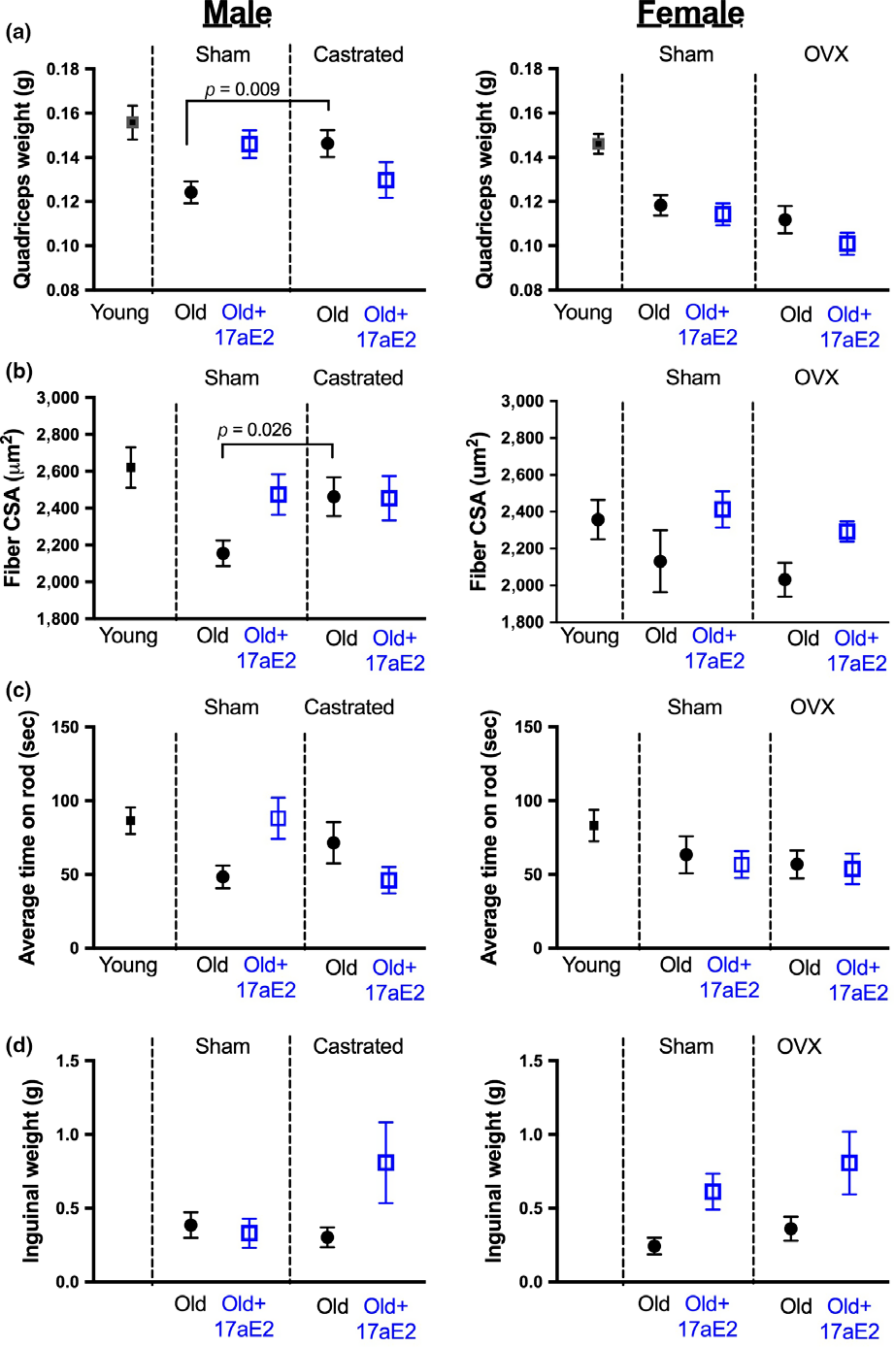
Functional and structural benefits of 17aE2 treatment in males are inhibited in males castrated prior to treatment. (a) Quadriceps weight (*N* = 9–16 per group), (b) gastrocnemius muscle fiber size (*N* = 8–16 per group), (c) rotarod capacity (*N* = 11–20 per group) and inguinal fat weight (N = 9–16 per group), in sham‐operated and gonadectomized males and females, examined at 25 months of age (24 m for rotarod capacity). Error bars represent *SEM* and *p* values are calculated from a Student's *t* test

We also assessed whether sex‐specific responses to 17aE2 in muscle structure and function were regulated by gonadal hormones, by comparing responses to 17aE2 in sham‐operated and castrated animals in cohort 1. Males castrated prior to 17aE2 treatment showed no increases in the weight of the quadricep muscle with 17aE2 treatment (Figure 5a: data for sham‐operated males are replicated from Figures 1, 2), indicating that the male‐specific response only occurs in males exposed to testicular production of hormones from 3 months of age. Castrated males have larger quadricep muscle weights than intact males on the control diet at 25 months, and 17aE2 treatment in intact males causes an increase in quadriceps weight to the level seen in untreated castrated males. This effect was the opposite of the castration effect in untreated animals on muscle weight seen in a subset of animals dissected at 12 months of age, where castrated males tended to have a lighter quadriceps (Supporting Information Figure S6), consistent with the short‐term effects of castration on skeletal muscle weight in adulthood (Jiao, Pruznak, Huber, Vary, & Lang, 2009). Similar to quadriceps weight, castrated male mice show no change in muscle fiber size with 17aE2 treatment, and again untreated castrated males have a larger skeletal muscle fiber CSA than that of equivalent untreated intact males (Figure 5b). This lack of responsiveness to 17aE2 treatment was also observed at the functional level, since castrated males showed no improvement in rotarod acceleration capacity with 17aE2 treatment (Figure 5c; intact male data are replicated from Figure 3). Intact and castrated males did not differ significantly in their rotarod scores in the untreated state. Females that were ovariectomized prior to treatment show similar treatment responses for each of these traits when compared to intact females (Table 1) and also showed an increase in inguinal fat mass similar to that observed in intact females (Figure 5d; surgery (OVX versus intact) by treatment interaction: *p* = 0.81).

### 2.7 Anti‐sarcopenic benefits of 17aE2 can be recapitulated by late‐life treatment (Cohort 2)

**Figure 6:**
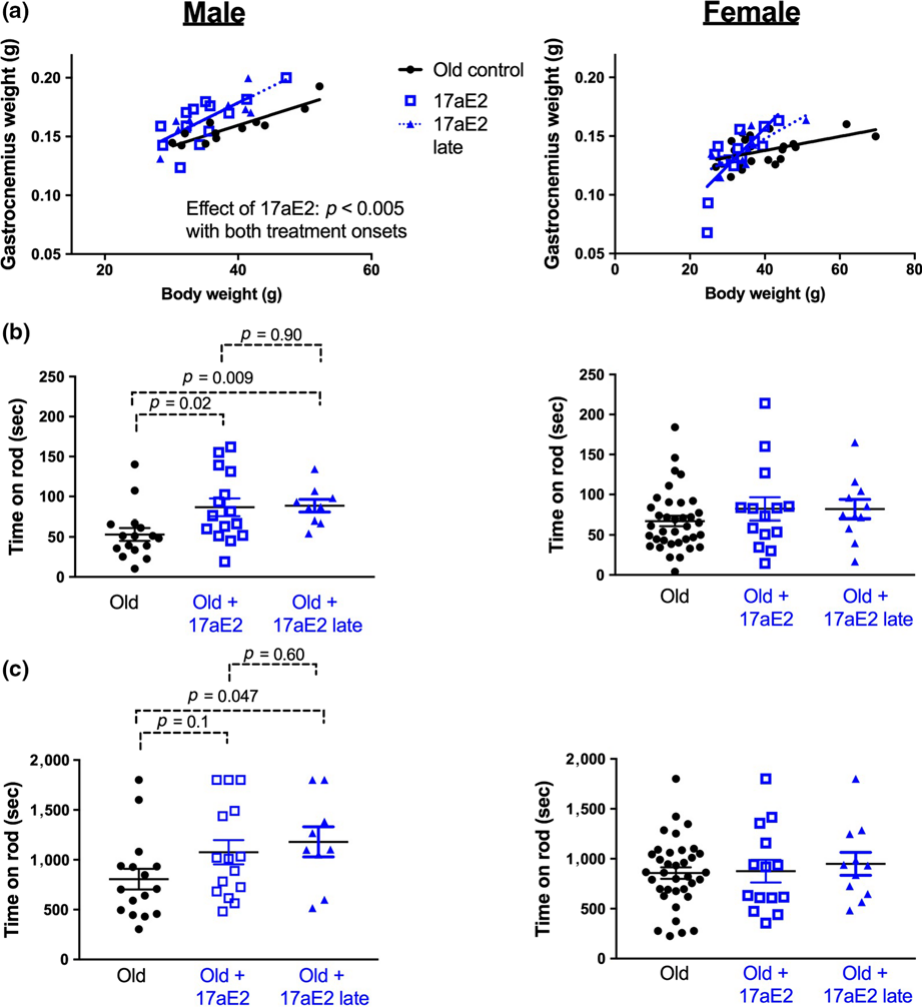
Benefits of 17aE2 treatment for muscle weight and rotarod function are recapitulated with treatment starting from 16 months. (a) The relationship between gastrocnemius muscle weight and body weight in 22‐month‐old mice on a control diet, 17aE2 from 4 months of age or 17aE2 treatment beginning at 16 months of age. (b) Rotarod acceleration and (c) endurance capacity in mice at 22 months. Each dot represents a value for an individual mouse (*N* = 9–36 per group). *p* values are calculated from an LSD post hoc test after establishing an overall group effect in a 1‐way ANOVA

In the second cohort of animals exposed to 17aE2 treatment, we evaluated a randomly selected subset of mice where treatment with 17aE2 began at 16 months of age. This allowed us to test whether the benefits of 17aE2 treatment for skeletal muscle aging and physical function could be recapitulated with a treatment beginning after middle age, an approach that may have advantages in some clinical settings if applied to humans. Male mice treated with 17aE2 from 16 months showed a larger gastrocnemius muscle weight at 22 months when compared to untreated animals, with this improvement being equivalent to that seen in individuals treated from 4 months (Figure 6a). The line of best fit shown in Figure 6a overlaps in male mice treated from these two different age points, making it difficult to discern the two lines in the figure panel. Female mice treated with 17aE2 from 16 months do not show a change in gastrocnemius muscle weight when compared to controls or animals treated with 17aE2 from 4 months.

This cohort of animals was also assessed for accelerating rotarod balance capacity. Like cohort 1, intact male mice treated with 17aE2 in cohort 2 again showed an improved rotarod capacity, with males treated from 16 months of age showing an equivalent improvement in performance to that seen with treatment from 4 months (Figure 6b). We further assessed the endurance capacity of mice by testing them at a lower and fixed rotation speed over a longer duration. Male mice treated with 17aE2 showed a longer endurance capacity than untreated males. The difference between control mice and mice treated with 17aE2 from 16 months was statistically significant, showing a benefit of late‐life treatment, whereas the difference between mice treated from 4 months and controls did not reach statistical significance (Figure 6c).

## 3 DISCUSSION

Pharmacological treatments that extend the lifespan of laboratory organisms deserve consideration as guides to interventions that could improve healthy aging in humans (Longo et al., 2015). A key criterion is that lifespan extension should be associated with improved physical function and health, which has not always been met when functional tests have been performed on long‐lived animals (Bansal, Zhu, Yen, & Tissenbaum, 2015; Richardson et al., 2015). In this study, we show that the lifespan extension observed with 17aE2 is associated with reduced age‐associated sarcopenia and improved late‐life physical function, benefits that can be gained even from a 6‐month treatment period beginning at middle age. However, these effects largely occur in a sex‐specific manner, matching the lifespan response seen with this treatment (Strong et al., 2016). Among the outcomes we tested, only elevations in skeletal muscle fiber size and altered body weight occurred to a similar degree in both sexes. The similar changes in body weight with 17aE2 in both sexes are particularly notable, since reductions in body weight with the onset of 17aE2 treatment have been linked to reduced feeding behavior as a consequence of actions at hypothalamic pro‐opiomelanocortin (POMC) expressing neurons (Steyn et al., 2018). The observation that body weight declines in both sexes with 17aE2, but functional benefits occur only in males, could suggest that the beneficial anti‐aging effects of 17aE2 are not purely a consequence reduced body weight and consumption of fewer calories, because we would expect this to benefit both sexes. Ultimately, normalization of food intake between controls and 17aE2 is required to definitively test this, either via a controlled feeding approach or by using a mouse model without functional POMC expression. Previous use of mice lacking POMC expression has shown some metabolic responses to 17aE2 can occur without changes in weight and feeding (Steyn et al., 2018), supporting the hypothesis that health benefits of 17aE2 are independent of reductions in calorie intake.

We used an untargeted primary metabolism screen to identify metabolic responses that are linked to the observed male‐specific elevations in skeletal muscle weight during aging. This demonstrated that 17aE2‐treated males show an increase in amino acids in quadriceps at 25 months. Notably female mice instead showed a decline in the abundance of some amino acids with 17aE2, although these females had muscle weights equivalent to control animals, indicating the relationship between 17aE2, muscle weight, and amino acid abundance is not bidirectional. In a previous study, we observed that this elevation in males (and decline in females) is not observed in quadriceps taken from animals with equivalent treatment sampled at 12 months of age (Garratt et al., 2018), indicating that effects of 17aE2 on the metabolome may differ depending on age, matching the age‐specific effects on muscle weight. To directly test whether the observed elevations in amino acid levels occur as a consequence of net alterations in protein synthesis or breakdown requires metabolic flux analysis, which was not possible in the long‐term aging studies designed here. However, our results suggest that 17aE2 induces changes in cellular processes involved in both protein synthesis and autophagy in adult mice, and these responses correspond to changes in amino acids in terms of reducing a sex difference observed in animals on a control diet. LC3BII levels were elevated in male mice treated with 17aE2, indicating altered autophagosome formation. In addition, total 4EBP1 abundance was reduced, without altered phosphorylation at sites activated by mTORC1. 4EBP1 is a translation initiation factor that when associated with eIF4E inhibits cap‐dependent translation. A reduction in abundance of 4EBP1 is expected to promote protein translation (Morita et al., 2013). Given the role of both autophagy and protein translation in the causal control of the aging in some species (Hansen & Rubinsztein, 2018; Steffen & Dillin, 2016), detailed studies that directly assess the effects of 17aE2 on autophagic flux, protein synthesis, and metabolomic flux, in both sexes, may provide an insight into sexually dimorphic cellular processes that modulate muscle mass and turnover during aging.

Our study was designed to provide causal insight into the endocrine mechanisms that underlie sex‐specific responses to 17aE2, and demonstrates that anti‐aging responses to this treatment are controlled by the presence of sex‐specific gonads. These results are consistent with our previous research showing that metabolic responses to 17aE2 in adult life are also dependent on gonadal hormones (Garratt, Bower et al., 2017; Garratt et al., 2018). We observed that skeletal muscle phenotypes induced by 17aE2 partly resemble those observed in untreated castrated males, and that castrated males do not respond to 17aE2 treatment, either in relation to muscle weight, physical function, or in their quadriceps metabolomic response. We have tested whether 17aE2 reduces circulating levels of testosterone in male mice but detected no observable decline in circulating testosterone levels in response to this treatment (Supporting Information Figure S7). However, testosterone is released from the testes in a pulsatile manner (Coquelin & Desjardins, 1982), making it difficult to accurately assess some measures of testosterone exposure without detailed kinetic studies. In a new cohort of C57BL/6J mice treated with 17aE2 from 3 months of age for seven weeks, we observed a highly consistent reduction in seminal vesicle weight (Supporting Information Figure S7), a reproductive organ that is very sensitive to circulating testosterone and its metabolite dihydrotestosterone (DHT), a more potent androgen in terms of binding affinity to the androgen receptor. This indicates that 17aE2 does reduce aspects of androgenic signaling, potentially explaining the resemblance of specific phenotypes to castrated males, and the lack of response in castrated males that already have low testosterone and DHT.

Although male mice treated with 17aE2 resemble castrated males in a set of skeletal muscle phenotypes, it is important to note that other aspects of sexual dimorphism normally controlled by gonadal hormones remain intact after 17aE2 treatment. Sex differences in the circulating concentrations of IGF1, leptin, and adiponectin persist with 17aE2 treatment (Garratt, Bower et al., 2017), in spite of the dependence of these sexual dimorphisms on gonadal hormones. The role of testicular and ovarian hormone release in control of sexual dimorphism is governed by sex steroids and their metabolites at various different levels, and at different developmental time points, and we speculate that adult‐onset 17aE2 treatment may interfere with steroidogenic actions at specific sites while leaving others intact. For example, 17aE2 is capable of suppressing 5‐alpha‐reductase activity in vitro, the main enzyme that mediates conversion of testosterone to DHT (Schriefers, Wright, Rozman, & Hevert, 1991). This would be expected to dampen signaling through the androgen receptor, including reducing the weight of seminal vesicle glands, without major feedback effects on other aspects of the hypothalamic–pituitary–gonadal axis (HPG) (Mahendroo, Cala, Hess, & Russell, 2001). Alternatively, 17aE2 could alter HPG axis feedback through binding to estrogen receptors in specific brain areas, which in male mice is partly mediated by negative feedback of the HPG axis after aromatization of testosterone to 17β estradiol (Fisher, Graves, Parlow, & Simpson, 1998). At the dose provided in this study, 17aE2 is capable of activating classical estrogen receptors (ER) in mice, as evidenced by the uterotrophic effects observed in ovariectomized female mice (Strong et al., 2016). Such stimulation in regions like the hypothalamus and pituitary could elicit negative feedback for the HPG axis, suppressing LH and FSH release and subsequent gonadal hormone release, while maintaining ER activation in the brain. In female mice, 17aE2 reduced the abundance of amino acids in muscle, and this female‐specific metabolomic effect was not seen in ovariectomized females, similar to ovarian hormone‐dependent female‐specific metabolomic responses in the liver (Garratt et al., 2018). This indicates that some female‐specific responses to 17aE2 are also dependent on ovarian hormones and would be consistent with the idea that 17aE2 interferes with the HPG axis in both sexes, but that this interference has observable beneficial health effects only in males. We have also shown that treating male mice with 17aE2 leads to a major male‐specific increase in hepatic estriol levels (Garratt et al., 2018), suggesting that 17aE2 may also be metabolized to additional estrogens in a sex‐specific way. Understanding the causal role of individual aspects of steroid signaling in aging, in central and peripheral tissues, may provide a major insight into the role of specific components of the HPG axis in aging in both sexes. This might ultimately lead to more precise pharmacological agents that provide the beneficial effects of sex‐steroid signaling for aging, while minimizing or ablating their negative effects on other aspects of health.

## 4 EXPERIMENTAL PROCEDURES

A detailed outline of all experimental procedures and statistical approaches is found in the supplementary information. UM‐HET3 mice were produced and maintained as previously described (Miller et al., 2014; Strong et al., 2008). Mice were given free access to water and were fed Purina 5LG6 after weaning. Mice were group housed in ventilated cages and were transferred to fresh cages every 14 days. Temperature was maintained within the range of 21–23°C. At 3 months of age, all animals in cohort 1 went through castration, ovariectomy or a sham procedure as previously described (Garratt, Bower et al., 2017; Garratt et al., 2018). Cohort 2 did not go through surgeries and had normal gonadal hormone production.

### 4.1 Diets: Cohorts 1&2

At 4 months of age, animals were randomly allocated to control or 17aE2 treatment. Animals in the control group remained on the 5LG6 diet, while animals allocated to 17aE2 had their diet switched to a food containing this drug at 14.4 ppm (see Strong *et al*. 2016). In cohort 2, a randomly selected subset of animals was maintained on the control diet until 16 months of age and then was switched to be treated with 17aE2 for the last 6 months of treatment.

### 4.2 Rotarod and grip strength tests

Animals in cohort 1 were tested for their ability to balance on an accelerating rotarod at 24 months of age. Animals were placed on the rotarod and the trial began with the spindle revolving at 5 revolutions per minute (RPM) and increased to 40 RPM gradually over a 5‐min period. The time at which the animal fell off the rotarod was used as a score, with each animal tested three times and the mean score used in analysis. The second cohort underwent the same testing protocol at 22 months of age. A subset of animals in cohort 2 were tested for grip strength using an EB1‐BIO‐GT3 grip strength meter with an EB1‐GRIP‐Mouse Grid. Subjects were removed from their cage by the base of the tail and suspended above the grid until their forepaws griped the grid. The tail was gently pulled in a horizontal direction away from the grid until the mouse released its grip. The maximal force was recorded. Each animal was tested six times with a 10 s rest between each. The mean of the six tests was used for analysis. All tests were conducted by an experimenter blind to treatment group and surgery status.

### 4.3 Euthanasia, tissue harvesting and processing

Animals were euthanized and tissues harvested during the morning after 18 hr of fasting. Tissues were weighed and then immediately frozen with liquid nitrogen and stored at −70°C unless otherwise stated. Deleted methodology for western blots, metabolomics, histology, and immunofluorescence is in the supplementary information.

## CONFLICT OF INTEREST

None declared.
