# Supplementary material for: 17‐α estradiol ameliorates age‐associated sarcopenia and improves late‐life physical function in male mice but not in females or castrated males
Source: Aging Cell. 2019 Feb 10;18(2):e12920. doi: 10.1111/acel.12920 (PMC6413653; doi:10.1111/acel.12920)
Supplement: Supplementary file 1 [file ACEL-18-e12920-s001.docx]

**Figure S1.** Quadriceps weights in male mice at 6, 12, 22 and 25 months of age. A. Quadriceps muscle weight in mice dissected at either 12 or 25 months of age, either on a control diet or 17aE2 (Cohort 1). B. Data from mice dissected at 25 months of age, in addition to a cohort of 6 month old mice that were dissected over the same period (Cohort 1). C. quadriceps muscle weight in mice dissected at either 22 months of age, either on a control diet or 17aE2 (Cohort 2). Each dot represents the body weight and quadriceps weight of an individual mouse.

**Figure S2.** Muscle fiber types and cross-sectional area of type 2a and 1 muscle fibers in gastrocnemius muscle of males and females treated with 17aE2. In A and B, each dot represents the mean value for an individual, with error bars showing S.E.M. In C, representative images are shown of type 2B muscle fibers, imaged in the posterior section of the muscle. In D, type 2a and type 1 fibers are shown in the anterior section of the gastrocnemius muscle, with red corresponding to type 2a fibers and grey type 1.

**Figure S3.** Cross sectional area of muscle fibers insoleus muscle of males and females treated with 17aE2. Each dot represents the mean value for an individual, with error bars showing S.E.M.

**Figure S4.** Amino acid responses to 17aE2 treatment in male and female mice. Additional amino acids are shown in figure 5. Sex by treatment interaction terms are shown in Table 2. P values are from a Student’s T-Test.

**Figure S5.** MTORC1 substrate phosphorylation status in quadriceps muscles of 17aE2 treated male and female mice.

**Figure S6.** Castration at three months of age has different effects on quadriceps weight at different ages. All mice shown are untreated and had been castrated or been through a sham surgery at 3 months of age. Error bars show S.E.M.

**Figure S7.** Testosterone levels and seminal vesicle weight in male C57BL/6J mice treated with 17aE2 for either 21 or 49 days from 4 months of age.

**Experimental procedures**

UM-HET3 mice were produced as previously described ([Miller et al., 2014](#_ENREF_3); [Strong et al., 2008](#_ENREF_6)). The mothers of the test mice were CByB6F1/J, JAX stock #100009, whose female parents are BALB/cByJ and whose male parents are C57BL/6J. The fathers of the test mice were C3D2F1/J, JAX stock #100004, whose mothers are C3H/HeJ, and whose fathers are DBA/2J. Mice in breeding cages received Purina 5008 mouse chow, and weaned animals were fed Purina 5LG6.

Mice were housed as previously described ([Miller et al., 2014](#_ENREF_3); [Strong et al., 2008](#_ENREF_6)) in plastic cages with metal tops, using ¼ inch corn-cob bedding (Bed O’Cobs, produced by The Andersons, PO Box 114, Maumee, Ohio). Mice were given free access to water, using water bottles rather than an automated watering system. Mice were housed in ventilated cages and were transferred to fresh cages every 14 days. Temperature was maintained within the range of 21–23 °C.

**Cohort 1: Surgical procedures**

At three months of age all animals in cohort 1 went through castration, ovariectomy or a sham procedure. All animals were anaesthetized by injection of 250mg/kg tribromoethanol, and given a single pre-operative injection of the analgesia carprofen, at 5mg/kg.

*Castration and sham castration*

After surgical preparation, an incision was made in the caudal end of each scrotal sac, the testicle was pulled through the incision by gentle traction, and the blood vessels, vas deferens and deferential vessels were clamped and sutured, before removing the testes. The incision was closed with tissue adhesive. For sham surgery, the testicles were exteriorized and then replaced in the scrotum, without being ligated or excised.

*Ovariectomy or sham ovariectomy*

After surgical preparation, an incision was made on the left side perpendicular to the vertebral column approximately midway between the iliac crest and the last rib. The ovarian fat pad was grasped and exteriorized. The pedicle under the ovarian blood vessels and fat pad under the ovary were grasped and crushed, the pedicle cut on the ovary side and the ovary removed, and the blood vessels tied with absorbable suture. The abdominal wall was closed with absorbable suture and skin was closed with staples. The procedure was then repeated on the opposite side. For sham ovariectomy, animals underwent the same surgical procedure, but the ovary and fat pad were exteriorized and replaced without being excised.

**Diets: Cohorts 1&2**

At four months of age, animals in different sibling groups were randomly allocated to control or 17aE2 treatment. Animals in the control group remained on the 5LG6 diet, while animals allocated to 17aE2 had their diet switched to a food containing this drug at 14.4 ppm. In cohort 2, a randomly selected subset of animals was maintained on the control diet until 16 months of age, and then were switched to treated with 17aE2 for the last 6 months of treatment.

Diets were prepared by TestDiet, Inc., a division of Purina Mills (Richmond, IN, USA). Purina 5LG6 was used as the control diet. 17aE2 was purchased from Steraloids Inc. (Newport, RI, USA) and mixed at a dose of 14.4 milligrams per kilogram diet (14.4 ppm) with Purina 5LG6. These methods followed those used by the NIA Interventions Testing Program.

**Rotarod and Grip strength tests**

*Rotarod*

Animals in cohort 1 were tested for their ability to balance on an accelerating rotarod at 24 months of age. Animals were tested on a Rotamex 5 (Columbus Instruments, Columbus, Ohio, USA), with a spindle dimension of 3 cm x 9.5 cm. Animals were placed on the rotarod and the trial began with the spindle revolving at 5 revolutions per minute (RPM) and increased to 40 RPM gradually over a 5 min period. The time at which the animal fell off the rotarod was used as a score, with each animal tested three times with 1-3 min rest between each trial, and the mean score used in analysis. The second cohort underwent the same testing protocol at 22 months of age, although a different rotarod apparatus was used (Ugo-Basile; model 47600 (Ugo-Basile, Gemonio, Italy). All tests were conducted by an experimenter blind to treatment group and surgery status.

*Grip Strength*

A subset of animals in cohort 2 was also tested for grip strength using an EB1-BIO-GT3 grip strength meter with an EB1-GRIP-Mouse Grid (Bioseb). Subjects were removed from their cage by the base of the tail and suspended above the grip until their forepaws griped the grid. The tail was gently pulled in a horizontal direction away from the grid until the mouse released its grip. The maximal force was recorded. Each animal was tested six times with a 10 sec rest between each. The mean of the six tests was used for analysis. Tests were conducted by a single experimenter blind to treatment group.

**Euthanasia, tissue harvesting and processing**

Animals were euthanized and tissues harvested during the morning after 18 hr of fasting. Tissues were weighed and then immediately frozen with liquid nitrogen and stored at –70 °C unless otherwise stated. For western blots, frozen quadriceps muscles were processed to generate whole-cell lysates, and equal amounts of protein were loaded for Western blot analysis. Antibodies and phospho-specific rabbit antibodies were purchased from Cell Signaling Technology (Danvers, Massachusetts) (total S6: catalog number 2217; pS6: 2211; p4EBP1: 2855; total 4EBP1: 9644; LC3B: 2775; β-Tubulin: 15115). Frozen quadriceps muscle from 25 month old mice was sent to the NIH West Coast Metabolomics Center, UC Davis, for untargeted analysis of primary metabolites. Twenty mg aliquots of frozen, homogenized tissue samples were extracted with -20 ºC cold, degassed mixture of isopropanol/acetonitrile/water (3:3:2, v/v/v), cleaned up after desiccation using 50% aqueous acetonitrile, dried again, and derivatized by methoximation and trimethylsilylation according to Standard Operating Procedures ([Fiehn, 2016](#_ENREF_2)). Briefly, metabolites were separated on a LECO Pegasus IV GC-TOF mass spectrometer using a 30 m rtx5 SilMS 0.25 mm x 0.25 mm column and a 10 m guard column. Data were acquired at -70 eV electron ionization voltage from 80-500 Da at 17 Hz with 1850 V MCP voltage. Data were processed by ChromaTOF 4.2 and then filtered and annotated by the BinBase database system ([Fiehn, 2016](#_ENREF_2)). The different trimethylsilylation products typically observed for amino acids in GC-TOF MS were summed for each compound to give one data point per amino acid. Data were normalized to the total intensity of all identified metabolites in each sample.

**Skeletal muscle fiber analysis**

*Hematoxylin and Eosin Stained gastrocnemius analysis: Cohort 1*

Gastrocnemius muscles from Cohort 1 were fixed in 10% buffered formalin, paraffin processed and embedded, sectioned, then stained with hematoxylin and eosin. Slides were evaluated on an Olympus BX-51 brightfield microscope at 20X magnification. The image was captured using an Olympus DP-70 high resolution digital camera. Each sample was imaged in three different areas of the gastroc (Medial head, Center, Lateral Head) to avoid regional biases. Images were then analyzed using ImageJ (Rasband, National Institute of Health, Bethseda MD). Cross-sectional area (CSA) was measured in µm² using the free-hand trace tool and total fiber count was calculated using the multi-point tool all within the software. Centralized nuclei of the muscle was determined by examining the three fields under a brightfield microscope and using a point-based system ([Sierra et al., 2013](#_ENREF_4)) (0=no centralized nuclei, 1= very few centralized nuclei i.e. 2-3, 2=some centralized nuclei, 3=many centralized nuclei). Atrophy of the muscle fibers was also determined using the same methods and a point-based system (0=no atrophied cells, 1= very few atrophied cells, 2=some atrophied cells, 3=many atrophied cells), with the total score across the three fields used for analysis. All analysis was conducted by one experimenter blind to sex and treatment group.

*Immunofluorescence analysis of fiber types: Cohort 2*

*Dissection, embedding, sectioning and staining*

Immediately after dissection muscles were placed in Fisher Healthcare Tissue-Plus O.C.T. Compound (Fisher Healthcare Houston, Texas) and quickly frozen in 2-methylbutane, then stored at -80°C. Muscles were cross-sectioned using a cryostat at a thickness of 10 µm, permeabilized using 0.3% Triton solution and a Mouse on Mouse kit (Vector Labs) used for blocking. Each skeletal muscle fiber type was identified using primary and secondary antibodies. Type I (slow-oxidative) fibers were detected using primary antibody BA-D5 DSHB Iowa (BA-D5) and secondary antibody Alexa Fluor-350 (Invitrogen Eugene, Oregon (A21140)), Type IIa (fast-oxidative) fibers were detected using primary antibody SC-71 DSHB Iowa (SC-71) and secondary antibody Alexa Fluor-555 (Invitrogen Eugene, Oregon (A21127)). Type IIb (fast-glycolytic) fibers were detected using primary antibody BF-F3 DSHB, Iowa (BF-F3) and secondary antibody Alexa Fluor-647 (Invitrogen Eugene, Oregon (A21235)).

*Imaging and Analysis*

Images were captured using a high resolution Axiocam camera system. Individual images were manually taken at 5X magnification. Within each gastrocnemius muscle three images were taken on the posterior side, and three images on the anterior side. The images from the anterior side of the gastrocnemius were subdivided into three regions: medial head, plantaris, and lateral head. Cross-sectional areas (µm²) of each fiber type within each image were analyzed manually using ImageJ, and the mean used for analysis.

**Statistics**

Statistics were carried out in SPSS version 22. In our analysis of effects of 17aE2 on specific parameters, we first focused on trait responses that occur in mice where gonadal hormone production was unaltered, i.e. mice that had only been through sham surgery (or all animals in cohort 2), since data on lifespan responses to 17aE2 was conducted in male and female mice without surgical manipulation ([Strong et al., 2016](#_ENREF_5)).

For each measured parameter we conducted a two factor ANOVA, using the general linear model function and a full factorial model, which included an effect of treatment (comparing control to 17aE2), an effect of sex (male or female) and an interaction between sex and treatment. In models where we were interested in controlling for effects of body weight we included body weight as a continuous covariate. If there was a significant interaction between sex and treatment we followed up by conducting a Student’s t-test to determine whether there was a significant effect of 17aE2 treatment within each sex. When testing for an effect of age we compared untreated animals, conducting a 2-way ANOVA that included an effect of age and an effect of sex and the interaction parameter. Data in Table 1 show P values for the age parameter, since there were no interactive effects with sex. When testing for the effect of gonadectomy on treatment responses within each sex, we included an effect of treatment, an effect of surgery (gonadectomized or not) and an interaction between surgery and treatment. Data was transformed logarithmically where necessary to meet parametric assumptions of associated tests.

*Analysis of quadriceps metabolites*

One hundred and forty seven metabolites were identified in the primary metabolism screen (Dataset submitted to the NIH Metabolomics Workbench). We first focused on metabolite responses that occur in mice where gonadal hormone production was unaltered.

All metabolite data was log-transformed; then for each metabolite, we fitted a two-way ANOVA model testing the effects of sex, treatment and the interaction between sex and treatment with data from sham operated males and females. By scanning through all interactions, we were able to identify metabolites that reveal significant treatment by sex interaction. Because multiple two-way interaction effects were tested across the dataset, we adjusted raw p-values with a False Discovery Rate (FDR) method ([Benjamini & Hochberg, 1995](#_ENREF_1)). For those metabolites found to show a significantly different response to 17aE2 according to sex, we then conducted subsequent Student’s tests within each sex to test whether 17aE2 lead to a significant increase or decrease in the metabolite of interest. For those metabolites that showed a significant sex by treatment interaction we subsequently tested whether male castration or female ovariectomy influenced the treatment response within either sex.

**References**

Benjamini, Y., & Hochberg, Y. (1995). Controlling the False Discovery Rate: A Practical and Powerful Approach to Multiple Testing. *Journal of the Royal Statistical Society. Series B (Methodological), 57*(1), 289-300.

Fiehn, O. (2016). Metabolomics by Gas Chromatography-Mass Spectrometry: Combined Targeted and Untargeted Profiling. *Current Protocols in Molecular Biology, 114*, 30.34.31-30.34.32. doi:10.1002/0471142727.mb3004s114

Miller, R. A., Harrison, D. E., Astle, C. M., Fernandez, E., Flurkey, K., Han, M., . . . Strong, R. (2014). Rapamycin-mediated lifespan increase in mice is dose and sex dependent and metabolically distinct from dietary restriction. *Aging Cell*, 10.1111/acel.12194. doi:10.1111/acel.12194

Sierra, E., Fernandez, A., de los Monteros, A. E., Arbelo, M., de Quiros, Y. B., & Herraez, P. (2013). Muscular senescence in cetaceans: adaptation towards a slow muscle fibre phenotype. *Scientific Reports, 3*. doi:10.1038/srep01795

Strong, R., Miller, R. A., Antebi, A., Astle, C. M., Bogue, M., Denzel, M. S., . . . Harrison, D. E. (2016). Longer lifespan in male mice treated with a weakly estrogenic agonist, an antioxidant, an α-glucosidase inhibitor or a Nrf2-inducer. *Aging Cell, 15*, 872-884.

Strong, R., Miller, R. A., Astle, C. M., Floyd, R. A., Flurkey, K., Hensley, K. L., . . . Harrison, D. E. (2008). Nordihydroguaiaretic acid and aspirin increase lifespan of genetically heterogeneous male mice. *Aging Cell, 7*(5), 641-650. doi:10.1111/j.1474-9726.2008.00414.x
