# Supplementary figures and images for: 17‐α estradiol ameliorates age‐associated sarcopenia and improves late‐life physical function in male mice but not in females or castrated males
Source: Aging Cell. 2019 Feb 10;18(2):e12920. doi: 10.1111/acel.12920 (PMC6413653; doi:10.1111/acel.12920)

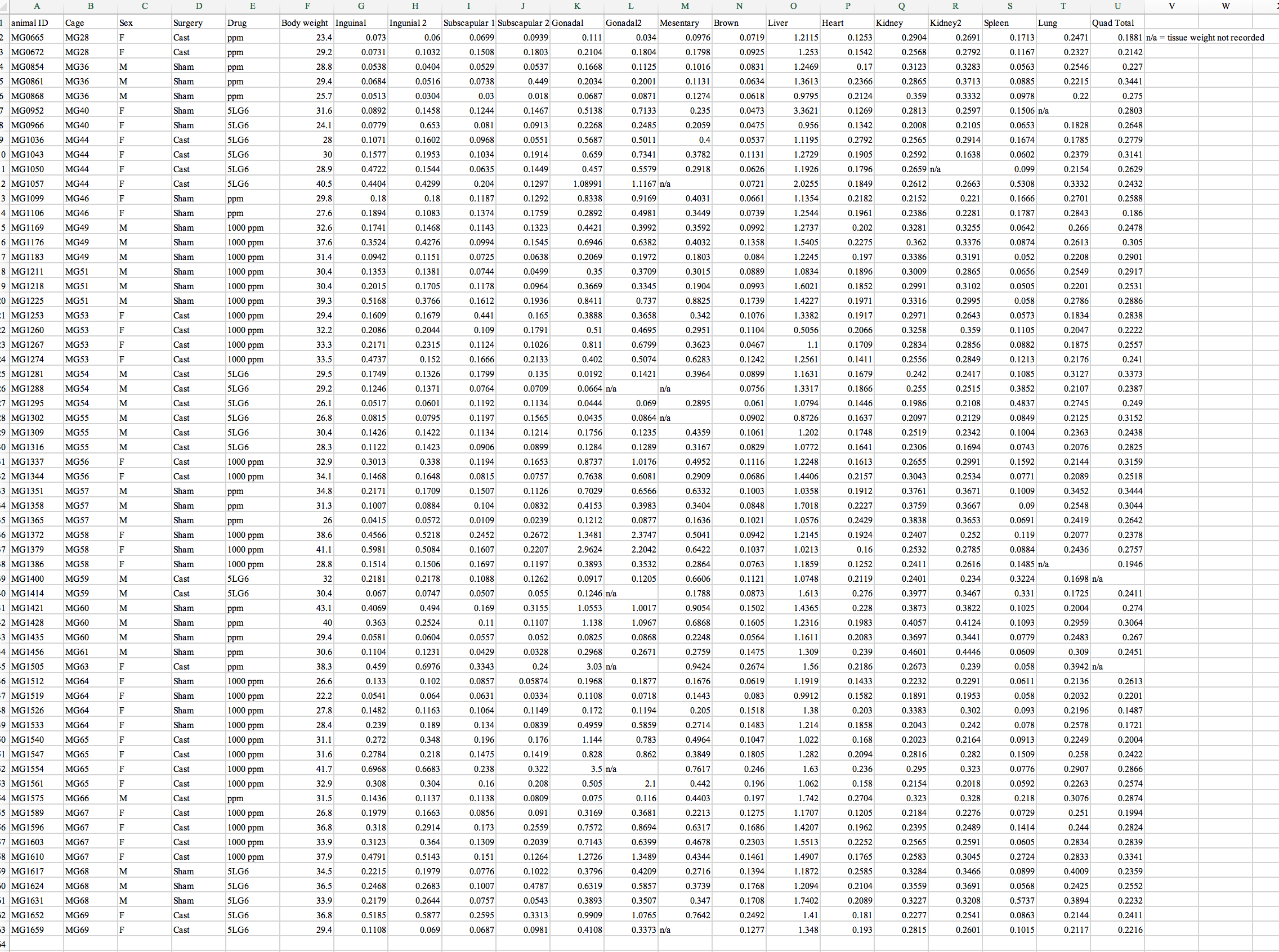


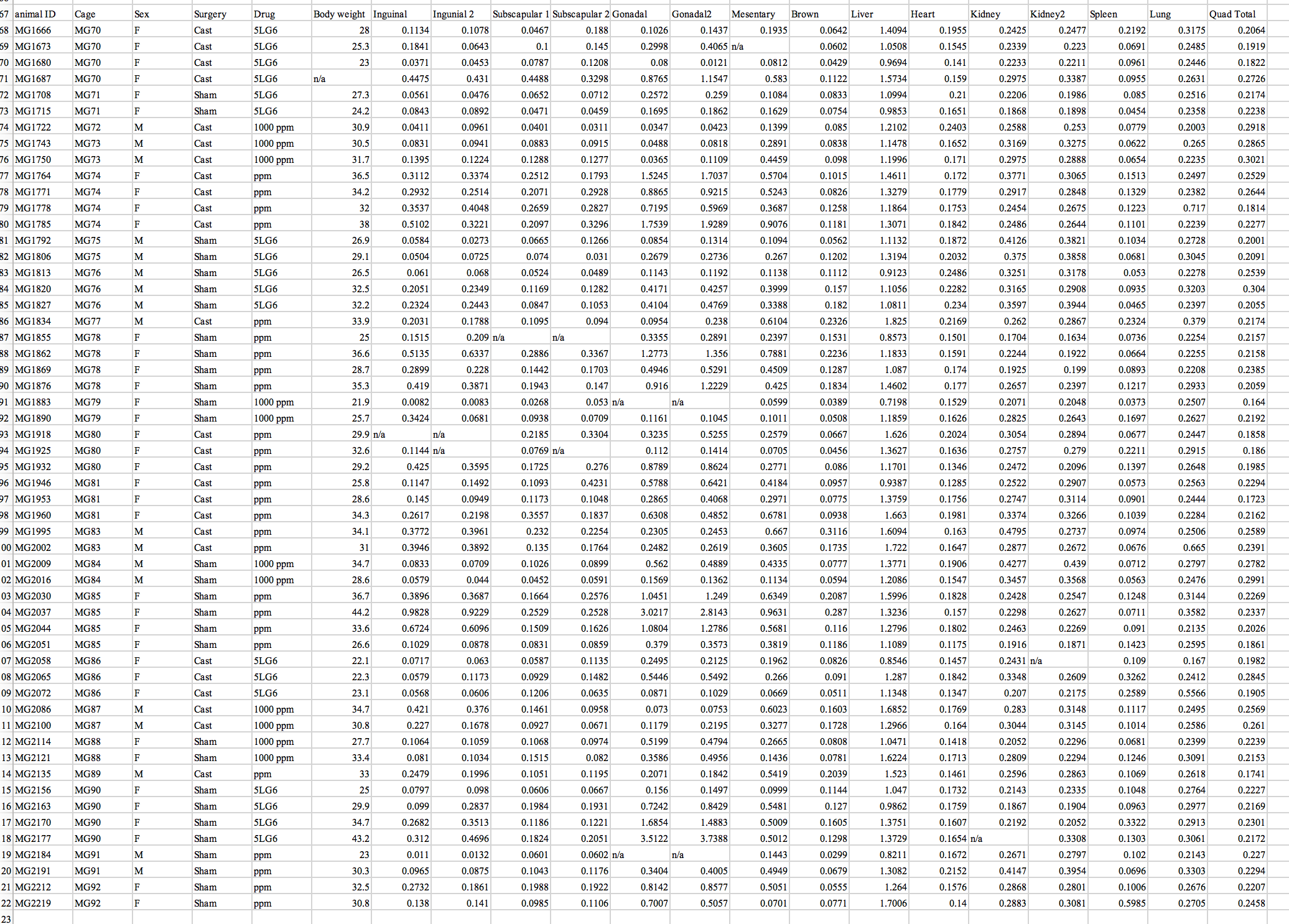


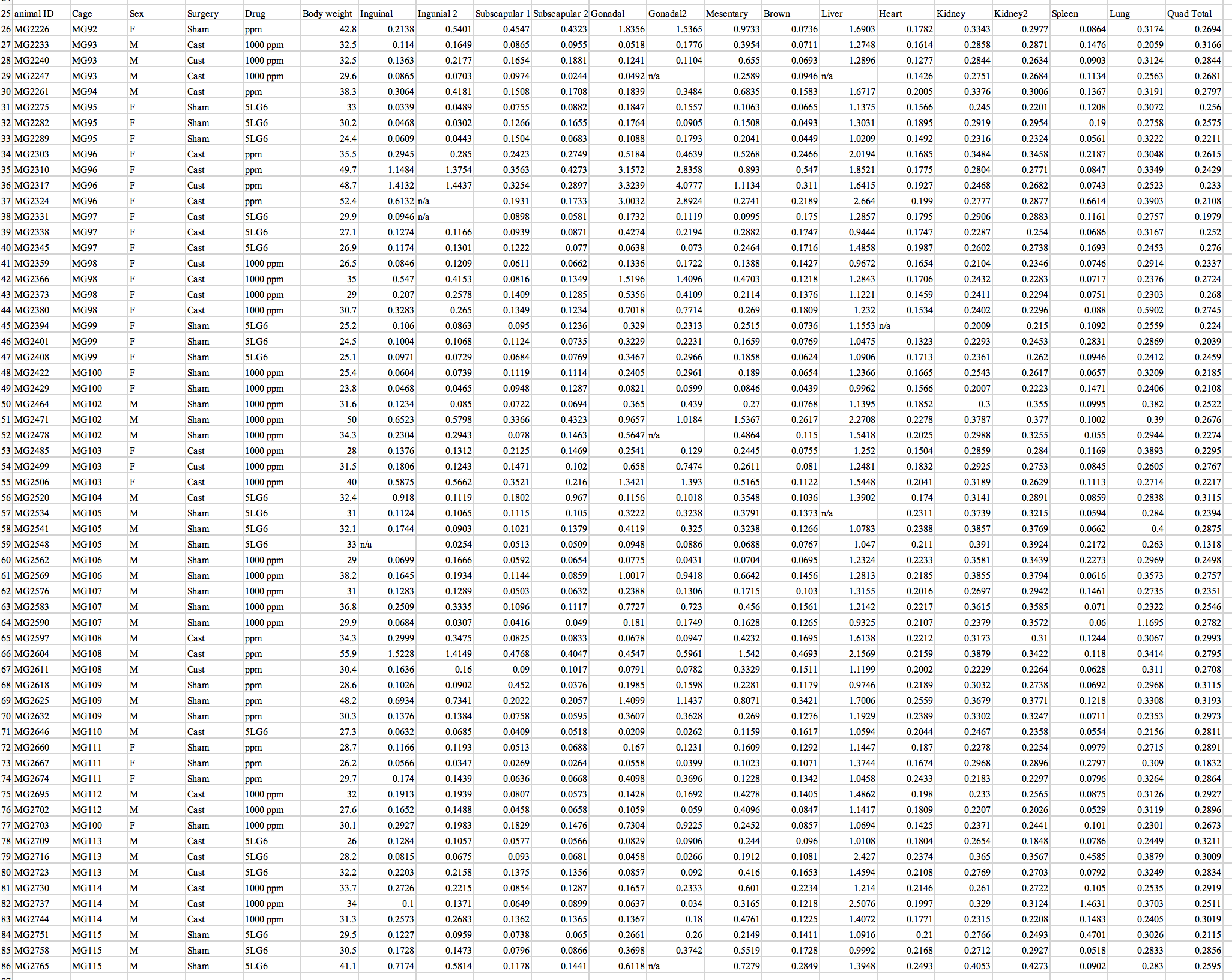

Supplement: Supplementary file 2 [file ACEL-18-e12920-s002.docx]
